# Dental Irregularities of the Native Races

**Published:** 1889-07

**Authors:** E. L. Townsend

**Affiliations:** Los Angeles, Cal.


ARTICLE II.

### DENTAL IRREGULARITIES OF THE NATIVE RACES.

BY E. L. TOWNSEND, D. D. S., LOS ANGELES, CAL.

"Are there any influences connected with civilization that are productive of degeneracy of the dental system? Is civilization to be charged with the frequent anomalies to be met with in the civilized man? Is decay of the teeth peculiar to civilization? Now, it is very clear that these questions cannot be answered intelligently, until the following question is answered : Are the dental systems of uncivilized races of men free from anomalies and all those diseases and malformations of the oral cavity so common to the civilized ?

"If we refer these questions to the general practitioner of either medicine or dentistry, the answer will be that the savage is exempt from these diseases and malformations, and that civilized man, owing to his artificial mode of living, requires artificial methods to support his organism ; that while his mental capacity has increased, it has been at the expense of his physical. When asked for proof of these statements, we are met with generalities ; statistics sometimes are quoted, but only from the side of civilization. Now, seeing that the savage has no asylums to investigate, no physicians or dentists to interrogate, that we could recognize as reliable, and no statistics to question, the conclusions drawn are quite *natural*, and being natural, as one-sided and very savage, but certainly not scientific."—*Dr. Patrick.*

These questions are of great importance, and why the savage has been deemed physically so perfect, is one of the conundrums of the age. It seems to be generally accepted, that the uncivilized and semi-civilized races are exempt from those dental ills which create so much discomfiture to civilized man ; that dental caries was but seldom found, and malformation a thing unknown. It is a pity that archæologists should have spent so much time in collecting things constructed by the savage races, and allowed the construction of the savage himself to pass unnoticed. If we had the percentage of dental anomalies found in the excavations, it would prove fully that the uncivilized man was subject to all the forms of dental irregularity that are found in the civilized races.

The savage, as he exists to-day, presents all the dental trouble found in his civilized brother. I have seen the stoical Sioux suffering the tortures of an ulcerated tooth, and his demeanor was much the same as would be a white man's under similar circumstances; and I venture the assertion that if an examination should be made of the various Sioux on their various reservations, not fifty per cent. of the adults would be found free from caries, and at least five per cent. would show irregularity of position; and these irregularities would present the same features as those found in civilized mouths. I have had fairly good opportunities for examining the reservation Sioux, both in Minnesota and Dakota; and the Klamath Indians of Oregon (physically the best Indians I have ever seen) have decayed teeth, and dental irregularities are more common with them than among any others I have noticed, excepting the Pueblos of New Mexico. One old fellow who was eternally lounging around Fort Klamath had a protruding under jaw, and having lost his back teeth the front ones had become abraded until the lower teeth passed the upper like a pair of shears, and presented as appalling an appearance as it was ever my misfortune to behold. A "four-bit" piece tempted the old rascal to bite into a piece of wax, and his condition is a matter of record. Many had protruding or V-shaped arches, but most of the irregularities consisted of the malposition of one or two teeth.

It was my good fortune to make the acquaintance of the first Indian agent of the Klamath reservation, a Mr. Applegate, who has spent upward of half a century among the Indians. He told me that they suffer from toothache and many other aches similar to the whites, and he said furthermore, that the females are not blessed in the way generally supposed, but that in many cases their children are born with as much pain and labor as come to the lot of civilized women. Many die, and only the more robust succeed in bearing a respectable sized family. The children, owing to the unprotected manner of life, fall victims to various diseases and only the best of them succeed in reaching adult age. This accounts for the physical development of those we see.

Many a gray-headed "49er" will tell you that the present generation is not as tough as they were, and point to the number of old argonauts, who are now the leading men on this coast, who are hale, hearty and seventy. This is a fact. They are the picked men of a picked crowd. In '49 it was no small undertaking to cross the plains; none but the best undertook it, and before the trip was ended they had been sorted time and again, until those who were left represented the best men, picked from the best and toughest race of men produced in this century; men bred and cradled under those influences best calculated to produce bone and muscle.

So it is with the Indian; the living denote the best, the weaker have succumbed. The reservation children show all the ills and deformities of civilized children. Whether this is because of the degenerating influences of civilization, or warmer houses and Government rations, is a matter yet to be determined; and all investigation of the Indian, relative to his dental equipment, will be hampered by the fact that those most likely to show deformity are buried and those in the most perfect state are to be found. The Pueblo Indians of New Mexico present at the present time the best opportunity for investigation of any of the Indian races. Their mode of life and habits tend to make them of especial interest to the dental student. They live in small villages or pueblos, and are to a limited degree agricultural. Their houses are by far the best made by Indians so far as I have seen, and their primitive modes of life should develop physically perfect men and women, if the common theories relative to the diet of man are correct. The squaws grind their grain between two stones and "lose none of the best of the grain," and the only effect on their teeth seems to be the excessive abrading of the crowns. The extra phosphates do not compensate for the extra grit in the flour; consequently the enamel wears away, the teeth become sensitive (just as sensitive as white men's teeth when abraded, if an Indian can feel as much), and hardly can an elderly Indian be found who does not know the pangs of an aching tooth.

Carious teeth are not uncommon and irregular teeth are frequently met with. I believe it no exaggeration to place the percentage at fifteen or twenty. I regret that I did not get models of their mouths, as it would have been an easy matter to have done so, for they are friendly and a ten-cent piece would open the mouth of the chief of any Pueblo village, and biting into wax for a monetary consideration would suit a Pueblo better than anything he now does.

Dr. F. M. Palmer of this city has made quite extensive explorations of the Rancherees on the islands off our coast, and has succeeded in making a very valuable collection of Indian relics, in fact one of the most complete in Southern California. He has resurrected upward of three thousand burials and has found several interesting specimens of dental deformity. He states that in fully fifty per cent. the teeth came together in direct antagonism; this appearance is undoubtedly due to the excessive abrasion occasioned by their mode of living. On his last trip he secured a very interesting specimen which shows conclusively that the same conditions existing in an uncivilized mouth will produce the same results as though the owner was civilized.—*Southern California Practitioner.*

